# The role of outcome expectancies and social support in adherence to nutrition counseling: perspectives of Emirati adults with diabetes

**DOI:** 10.3389/fpubh.2026.1805632

**Published:** 2026-04-16

**Authors:** Habiba I. Ali, Amal I. Al Harbi, Maitha Alnahdi, Mahra S. Alshamsi, Mariam R. Aldhaheri, Shamma M. Al Meqbaali, Meera A. Aldahmani, Nayab Fatima, Hafiz Muhammad Shahbaz, Moath F. Bataineh

**Affiliations:** 1 Department of Nutrition and Health, College of Medicine and Health Sciences, United Arab Emirates University, Al Ain, United Arab Emirates; 2 Imperial College London Diabetes Centre, Al Ain, United Arab Emirates; 3 Center for Non-Communicable Diseases, Karachi, Pakistan

**Keywords:** diabetes management, dietary adherence, nutrition counseling, outcome expectancies, qualitative interviews, social cognitive theory, social support, United Arab Emirates

## Abstract

**Objective:**

Diabetes represents a major public health burden in the Middle East and North Africa (MENA) region. However, limited research has explored patients’ lived experiences and perspectives on diabetes management, particularly nutrition, within the Arab region. This study examined factors influencing adherence to dietitian-led counseling among adults with diabetes in the United Arab Emirates (UAE), with a focus on social support, outcome expectations, and patient suggestions to enhance motivation for dietary adherence.

**Methods:**

A qualitative study using semi-structured individual interviews was conducted with 44 adults with diabetes attending a diabetes management clinic in the UAE. Audio-recorded interviews were transcribed and analyzed using NVivo-12. Inductive thematic analysis guided by Social Cognitive Theory (SCT) was used to identify key concepts related to outcome expectations and social support. Participants’ suggestions for improving motivation to seek nutrition advice from dietitians were also explored.

**Results:**

Four main themes emerged from the analysis: (1) positive expectations, (2) negative expectations, (3) enablers and motivators, and (4) participant suggestions. Positive outcome expectations, including improved health, better glycemic control, and weight management, motivated adherence to dietary advice. Social support from family members, friends, and healthcare professionals facilitated adherence and attendance at dietitian consultations. In contrast, misinformation, low awareness of the role of dietitians in diabetes management, and skepticism toward nutrition advice acted as barriers. Participants encouraged others with diabetes to consult dietitians and adopt healthier lifestyle behaviors.

**Conclusion:**

Enhancing culturally appropriate social support and addressing informational barriers may improve dietary adherence, increase engagement in dietitian-led counseling, and improve nutrition-related diabetes outcomes.

## 1 Introduction

Diabetes Mellitus (DM) is recognized as a significant global public health challenge, requiring intensified efforts to manage and prevent it (1). Globally, diabetes remains a leading cause of disability and mortality (1). According to the International Diabetes Federation (IDF), 10.5% of adults aged 20–70 years were living with diabetes in 202, projected to increase to 12.2% in 2024 (2).

The Middle East and North Africa (MENA) region had the highest comparative prevalence of diabetes among adults aged 20–79 years in 2021, at 18.1%, a figure projected to rise to 20.4% by 2045 (2). However, prevalence varies across countries within the region. Countries in the Arab Gulf report particularly high rates, with Kuwait having one of the highest comparative prevalence rates at 24.9% (2). In the United Arab Emirates (UAE), the age-adjusted prevalence of diabetes among adults aged 20–79 years was estimated at 16.4% in 2021 (2).

Several factors contribute to the high burden of diabetes in MENA countries, including aging populations, reduced physical activity, excess energy intake, unhealthy dietary patterns, and obesity (3). In addition, countries in the Gulf region share similar cultural and socioeconomic characteristics, such as lifestyles, dietary habits, income levels, language, and religion, which may influence health behaviors and disease risk (4). In regions with high rates of consanguinity, genetic and epigenetic factors may also contribute to the elevated prevalence of diabetes (5).

Effective diabetes management is critical given the high and increasing prevalence of the disease globally and in the MENA region. Uncontrolled diabetes can lead to severe complications, including nephropathy, cardiovascular disease, and retinopathy. Comprehensive diabetes self-management strategies, including dietary modifications, physical activity, medication adherence, and regular glucose monitoring, are therefore essential for long-term health outcomes (6, 7). Among these strategies, dietary management plays a pivotal role as diabetes is an inherently metabolic disorder (8–11). Medical nutrition therapy (MNT), delivered through structured dietitian-led counseling and motivational interviewing, has demonstrated significant improvements in glycemic control, body composition, and overall health outcomes (12–14). Despite the proven benefits of dietitian-led interventions, adherence to dietary recommendations remains a significant challenge for many patients, due to a combination of personal, cultural, and environmental factors.

Adherence to dietary counseling is influenced by cultural beliefs, perceived barriers and motivations, past experiences, and current life circumstances (15, 16). Enablers to dietary adherence include a good understanding of the importance of nutrition counseling and acceptance of dietitians as experts in dietary management (17–19). On the other hand, misconceptions about diet and dietitians build reluctance among patients to seek dietary advice (20). Social support is also crucial in promoting behavior change and adherence to nutrition counseling (21, 22). Therefore, for healthcare interventions to be effective, these facilitators and barriers must be addressed with an in-depth understanding of patients’ capacity for long-term behavior change (23). Such an approach will help prioritize the necessary areas of attention for redesigning or modifying existing clinical protocols and fostering future interventions for people living with diabetes (24).

In this context, Social Cognitive Theory (SCT) (25) offers a robust framework for exploring the determinants of an individual’s adherence to dietary recommendations. SCT proposes that behavior is influenced by interactions among personal, behavioral, and environmental factors (25, 26). Key constructs include self-efficacy, self-regulation, outcome expectancies, and social support (25, 26). In health promotion, according to SCT, individuals’ expectations regarding the outcomes of their actions and the social support they receive from family, peers, and healthcare providers can significantly influence their self-motivation and adherence to dietary guidelines (22, 27, 28).

Since SCT constructs are closely linked to individuals’ perceptions, beliefs, and lived experiences, qualitative research is especially suited to exploring factors influencing dietary adherence. Qualitative research enables a deeper understanding of how people with diabetes’s past experiences, beliefs, and attitudes, factors which might be overlooked by quantitative approaches (29–31). This perspective is particularly important in the cultural context of the UAE, where limited research exists on how socio-cultural norms, family dynamics, and accessing healthcare services impact diabetes self-care. The unique effects of these factors on diabetes self-management warrant further investigation.

We previously reported patients’ perspectives on diabetes nutrition management within the framework of the SCT’s self-regulation and self-efficacy constructs (32). Therefore, in this study, we primarily focused on identifying factors influencing diabetes nutrition management within the context of the SCT’s social support and outcome expectancies constructs. Understanding the motivating factors and social support for diabetes nutrition management could improve nutrition counseling services provided to patients with diabetes in the UAE. Moreover, understanding this topic from the patients’ perspective is crucial. Thus, this study aimed to explore the perspectives of individuals with diabetes attending a specialized diabetes management clinic in the UAE regarding factors influencing their adherence to dietitian-led nutrition counseling sessions. The study also sought the participants’ suggestions for enhancing patients’ motivation to apply dietitians’ advice in diabetes management.

## 2 Methodology

### 2.1 Study design

This study employed a qualitative design using semi-structured individual interviews and inductive thematic analysis guided by constructs of Social Cognitive Theory (SCT) to explore patients’ perspectives on diabetes nutrition management. Interviews were conducted with adult patients attending a large, specialized diabetes treatment health clinic in Al Ain, United Arab Emirates. Qualitative methods are well-suited for exploring experiences, perceptions, and attitudes related to health behaviors, allowing the investigation of issues that cannot be measured through quantitative approaches (33). Such an approach improves our understanding of human emotions and experiences and is useful in everyday situations. Participants’ perspectives, opinions, and attitudes about their diabetes self-management techniques in relation to diet, and their confidence in managing their diabetes effectively, were found to be best obtained through qualitative research methodologies (34, 35). These topics are especially pertinent when examining how patients view the nutrition counseling services offered by dietitians (36). This study was conducted as part of a larger study investigating patients’ perspectives on adherence to dietitian nutrition recommendations and their attendance at dietitian nutrition counseling sessions (32). The study site was an outpatient facility specializing in diabetes treatment and education, located in Al Ain, United Arab Emirates. The center provides comprehensive health services, including nutrition counseling by licensed dietitians. The research project was carried out from October 2022 to March 2023.

### 2.2 Theoretical framework

Medical nutrition therapy (MNT) provided by dietitians plays an important role in improving glycemic control and overall health outcomes in individuals with diabetes (37). In the United Arab Emirates (UAE), dietitians are part of multidisciplinary teams working in specialized diabetes management clinics. Despite the recognized benefits of dietitian-led counseling, attendance at dietetic consultations is often suboptimal, and limited research has explored the factors influencing patients’ engagement with these services. Poor attendance at dietary counseling sessions may compromise effective diabetes self-management, which requires sustained behavioral changes. To better understand these factors, this study applied selected constructs from Social Cognitive Theory (SCT) (25), specifically outcome expectancies and social support, to examine patients’ perceptions of influences on their adherence to dietitian-led nutrition counseling in a specialist diabetes management clinic in the UAE.

### 2.3 Ethical considerations

The Imperial College London Diabetes Centre Research Ethics Committee provided ethical permission for this study (approval #: IREC 080). All study methods were carried out per the guidelines and regulations of the Imperial College London Diabetes Centre Research Ethics Committee (approval date: August 22, 2022). In line with the approved protocol, strict ethical guidelines were followed throughout the study to protect participants’ rights, well-being, and the safety of their health-related information. All participants received comprehensive information about the study’s goals and methods, as well as the possible risks and benefits of participation, prior to their involvement. Participants gave their informed consent verbally and in writing. In order to ensure reliable data collection, participants also agreed to have their interviews audio-recorded on the clinic’s premises. The privacy and confidentiality of the study’s data were rigorously maintained.

### 2.4 Participant recruitment

Details of Participant recruitment were previously described (32). Adult patients with diabetes who were receiving care at a diabetes management clinic in Al Ain, UAE, were recruited for the study. Males and females aged 19 years or older with type or type 2 diabetes who were receiving diabetes management services at the clinic were eligible to participate. The study did not include patients with other types of diabetes. Purposive sampling was used to recruit participants with varying durations of diabetes (e.g., recently diagnosed to longer diabetes duration) to capture diverse perspectives, including those who had visited a dietitian for nutrition counseling in the past six months and those who had not. The purposive sampling technique is used in qualitative research to recruit individuals who meet pre-established criteria (38). Patients attending the diabetes clinic were invited by senior dietetics students to participate in a single individual interview. The number of patients who declined to participate was not recorded; however, time constraints, especially before the physician appointments, were noted as the main reason for non-participation.

Although both UAE nationals and non-nationals were eligible for inclusion, all patients present in the clinic waiting area during the recruitment periods were Emirati nationals, as the center primarily serves Emiratis. Therefore, all participants included in the study were UAE nationals.

### 2.5 Data collection

A single, one-on-one interview was conducted with adults with type 1 or type 2 diabetes from October 3, 2022 to March 24, 2023. The interview discussion guide focused on three main areas: (1) perceived benefits from dietitian nutrition counseling visits (2 key questions), (2) perceived social support for nutrition-related diabetes management (3 key questions), and (3) participants’ own suggestions for motivating patients to attend dietitian nutrition counseling sessions (1 question) (Supplementary material 1). The development of the guide was informed by two main constructs of SCT’s (25) outcome expectancies and social support. It was developed by the first, third, fourth, fifth, and sixth authors and validated by the second author. The discussion guide was initially developed in English and subsequently translated into Arabic by senior-year undergraduate students majoring in dietetics who are bilingual in Arabic and English (the third, fourth, fifth, and sixth authors). An Arabic-speaking senior research team member (second author) reviewed the translation to ensure its accuracy and equivalence. The questionnaire was then piloted with four participants who had received nutrition counseling at the clinic, resulting in minor revisions to simplify terminology.

Individual semi-structured interviews were conducted with patients who had consented to participate in the study. Two female undergraduate students (the third and sixth authors), both senior nutrition and dietetics majors, conducted the interviews after completing the first author’s training in qualitative data collection. Neither facilitator had prior interactions with the participants other than inviting them to participate in the study. One student led the discussion during each session while the other observed silently and managed the audio recording of the interview. None of the dietitians working in the clinic was involved in or aware of the research project.

Before the interview, participants were briefed on the study’s purpose and reassured that their data would remain confidential. They were also reminded of their right not to answer any questions they did not want to answer or to discontinue the session at any time. They were asked to avoid personal identifying information, such as their family names and names of health professionals involved in their care. The facilitator emphasized the researchers’ independence from the clinic and encouraged them to share their opinions openly.

All interviews were conducted in Arabic, the participants’ native language, and were audio recorded. A discussion guide was used to minimize bias, but additional questions were asked when necessary to obtain further details or clarifications. The interviews were held in a private office within the health center. Each interview lasted 15–20 min to cover questions on outcome expectancies and social support.

Audio recordings of the interviews were translated into English for analysis. Participants’ medical records provided supplementary information, including their age, duration of diabetes, and whether they had visited a dietitian in the previous six months. As the interviews progressed, the audio recordings were reviewed by the third, fourth, and fifth authors to identify areas requiring further probing. Preliminary analysis conducted after the first 23 interviews informed additional probing in subsequent interviews, particularly to explore negative expectations regarding dietitian-led nutrition counseling. This process also guided the targeted recruitment of participants who had visited the dietitian in the past six months. Recruitment continued until no new information related to SCT constructs of outcome expectancies and social support emerged, indicating data saturation at 44 interviews (39).

### 2.6 Data analysis

The data were analyzed using inductive thematic analysis (40), a widely used qualitative method suitable for systematically identifying patterns and themes within participants’ narratives and experiences, with NVivo 12 (QSR International) used to organize the interview transcripts. A continuous comparative approach was employed, in which each item of data was systematically compared with the rest to identify themes (41). This text-based thematic analysis ensured a robust and iterative process throughout the data analysis. Data collection and analysis occurred concurrently, creating a dynamic workflow. As interviews were completed, preliminary codes and categories were identified from the data. This iterative approach allowed researchers to refine the interview questions based on early findings (29) and target patients who had visited the dietitian in the past six months, ensuring a more comprehensive exploration of emerging themes as the study progressed. Following qualitative research principles, these initial analyses informed subsequent probing questions.

The analysis involved categorizing codes into categories and themes guided by the Social Cognitive Theory constructs of social support and outcome expectancies related to dietary counseling for diabetes management, forming thematic paths that linked participant statements to the final themes. Two researchers (the third and sixth authors) independently coded the transcripts, while two additional researchers (the fourth and fifth authors) reviewed the codes and emerging themes to assess their validity. The research team held several meetings to compare coding and discuss emerging interpretations. Any discrepancies in coding or theme development were discussed and resolved through consensus, with input from all team members to ensure consistency and analytical rigor.

### 2.7 Quality assurance

To maintain data integrity, the two facilitators who conducted the interviews transcribed the audio recordings verbatim, in Arabic. The facilitators themselves subsequently translated the Arabic transcripts into English. To enhance the accuracy and consistency of the transcripts, two additional team members independently reviewed the transcripts against the original audio recordings. In addition, team members who were not involved in conducting the interviews independently evaluated a random sample of 20 translated transcripts to ensure objectivity and verify translation accuracy. When clarifications were needed, the facilitators revisited the relevant audio recordings to confirm specific participant statements. The first author, with extensive expertise in qualitative research, reviewed the coding of the emerging themes and sub-themes to ensure analytical rigor. Participant quotes have been included in the results section to provide depth and reinforce the credibility of the findings. The study adhered to the Consolidated Criteria for Reporting Qualitative Research (COREQ) checklist (42) to maintain transparency and comprehensive reporting of the qualitative research process (Supplementary material 2). Collectively, these measures enhanced the validity and reliability of the study, ensuring clarity in the methods used.

## 3 Results

### 3.1 Participants

Forty-four participants (52.3% male) participated in the interview. Their ages ranged from 34 to 72 years, with the duration of diabetes varying between 1 and 38 years. Only one participant was not taking hypoglycemic medications (oral or injectables). 19 participants reported not having visited a dietitian during the 6 months preceding the interview.

### 3.2 Themes and sub-themes

The study explored patients’ perspectives on nutrition counseling in the context of SCT constructs, including outcome expectancies and social support. In addition, participants’ suggestions were sought on ways to enhance their motivation to implement dietitians’ recommendations for diabetes management.

**Figure 1:**
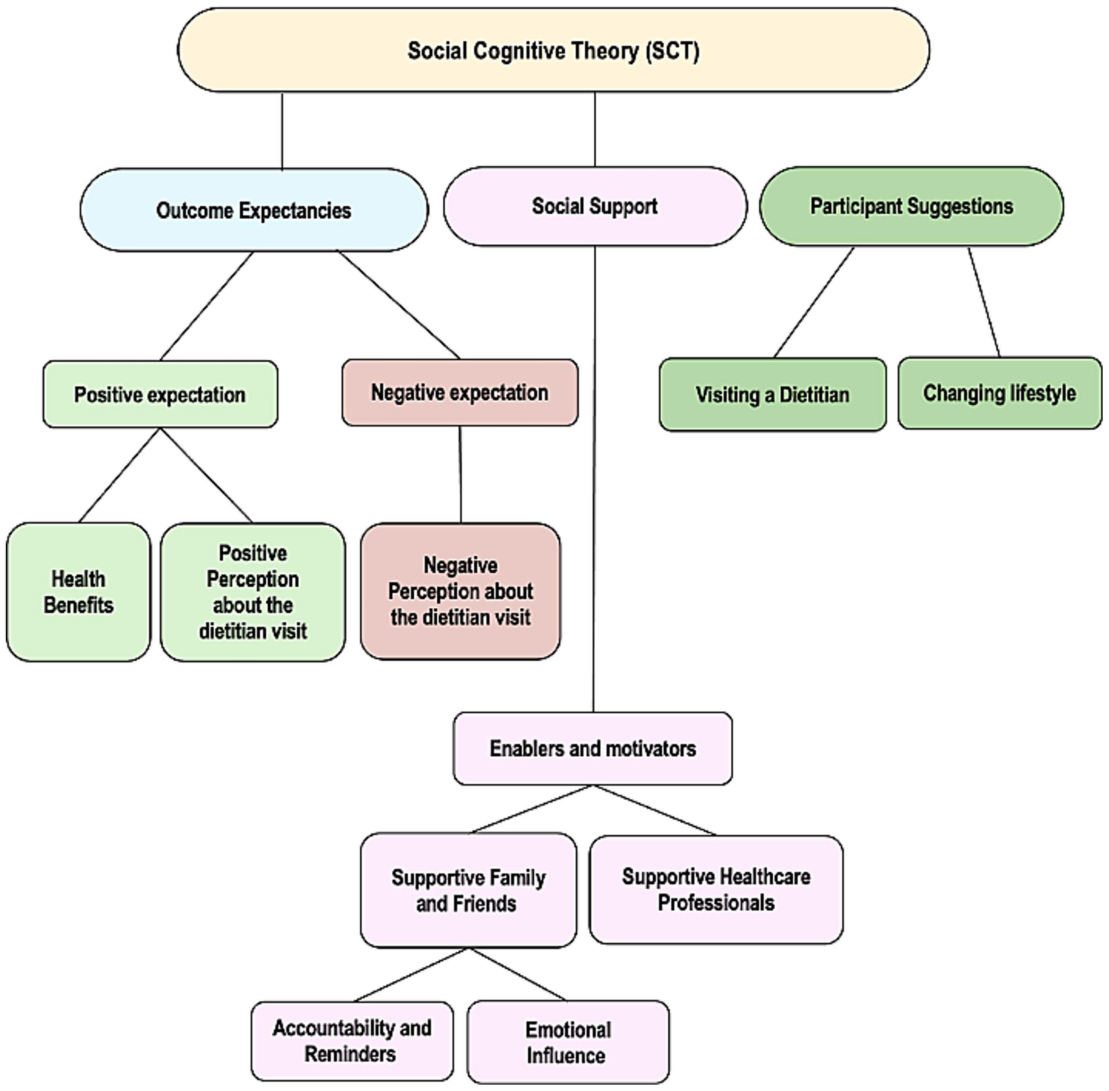
Themes and sub-themes emerging from the interviews.

Four primary themes emerged from the analysis: (1) positive expectations, (2) negative expectations, (3) enablers and motivators, and (4) Participant suggestions. Figure 1 shows the primary themes and sub-themes that emerged from the interviews. The first two themes align with the SCT’s outcome expectancies construct, and the third theme with SCT’s social support construct. The fourth theme is participant suggestions.

#### 3.2.1 SCT outcome expectancies

Participants shared diverse perspectives regarding their expectations of nutrition counseling services. The two primary themes related to the SCT outcome expectancies construct that emerged from the transcript analysis were positive expectations and negative expectations. While some patients anticipated positive outcomes from attending nutrition counseling sessions, such as improved weight management and regulated blood glucose levels, others remained skeptical about the potential benefits of dietetic services. Patient testimonials emphasize these varying viewpoints.

##### 3.2.1.1 Theme 1: positive expectations

Interviewing patients enabled the gathering of their thoughts, attitudes, and beliefs about the role of a dietitian as well as their overall perceived outcomes of nutrition counseling from dietitians. The two sub-themes that emerged from the positive expectations were health benefits, including improved diabetes management and weight management, and positive perceptions about dietitians.

###### 3.2.1.1.1 Sub-theme 1.1 health benefits

Participants in the study perceived that there were certain health benefits to be gained from attending a dietitian appointment, including improved blood glucose control and weight management. The most commonly expected outcome of visiting a dietitian, shared by multiple patients, was that the dietitian would provide advice to help reduce blood glucose levels.

The following quotes from participants illustrate the benefits of a visit to the dietitian and the perceived outcome expectancy cited by the participants:

*“It is important to visit a nutritionist, and because some people are not familiar with the diet, a doctor [dietitian] helps them as long as she is understanding and an expert in her field … During my visit today, I discovered things I did not know about. However, I am an educated person. I am an engineer and totally ignorant about health and food, as she is ignorant about my field. So, it is necessary to visit her to find out what is beneficial for the body and what is harmful because she is an expert in that. I don’t have diabetes, but I constantly check to find out my blood sugar level. From the beginning, a person should maintain his body’s health, eat, and exercise for at least half an hour per day. They should reduce sugars, starches, and fats from fatty things [foods], follow up with specialized doctors until the disease is detected from the beginning or even before it occurs.”* [Participant # 3].

*“Yes, she is the one who helped me honestly in reducing weight, regulating diabetes, and arranging my eating, because before, I used to eat anything and things that I did not know, and they explained to me the good things from the food.”* [Participant # 21].

*“She advises me about food to eat properly, healthy eating. Because I do not know the food we eat, now they say to us eat this, eat vegetables; before what we found [available], we ate, we do not know, we do not know that sugar is harmful.”* [Participant # 26].

Not only did patients expect to learn how to reduce their blood sugar levels, they also expected to learn how to deal with hypoglycemia appropriately: *“I follow up with her [the dietitian] because of the obesity as well, so she tells me to eat this and avoid this, and to eat a small percentage of this, so a specialist follows up with me about the weight and the amount of sugar in the blood, and she tells me to eat to sweets, and I must eat so as not to deprive myself. She gives me many tips for avoiding sudden sugar drops.”* [Participant # 23].

###### 3.2.1.1.2 Sub-theme 1.2 positive perceptions about visiting a dietitian

Interview participants believed that the role of a dietitian is essential and expected a positive outcome from visiting a dietitian. One of the participants stated, *“It is useful and important to visit a nutritionist and benefit from it. The benefit of visiting a nutritionist is weight improvement; she gives the patient suggestions and advice about what is useful and harmful to health and the right amount, and advises her to exercise as well.”* [Participant # 1].

Participants recognized dietitians’ expertise in delivering essential information and guidance on diabetes management, as reflected in the following statement: *“Yes, it helps him [patient], and a visit is a must. We must visit the dietitian, even if it is once, twice, or three times. It is not necessary for each visit [to the clinic]. If you are knowledgeable about a specific topic, you would be able to help me. If I have specific knowledge, each one will complement the other. The dietitian is sure that her studies are her specialty and that her information is more than anyone else’s, so when you tell her you can get certain benefits from her, she will benefit you. Yes, yes, I confirm that her words to the patient are correct and the nutritional program is correct.*” [Participant # 25].

Participants appreciated the role of dietitians in clarifying misconceptions and imparting knowledge: “*Sure. The dietitian knows things we do not; today, I learnt for the first time that vegetables contain high levels of carbohydrates. I did not know, okay; I mean, I know they contain carbohydrates, but I didn’t know they were this high. I mean, like potatoes, potatoes we know that they contain carbohydrates, but Pumpkin I did not know that they contain high carbohydrates, so the day you go to the nutritionist, he will explain this to you, and you will know.”* [Participant # 36].

Furthermore, patients indicated that a dietitian’s advice is a source of motivation and accountability, helping them to reach their goals: “S*he gives advice and instructions… she gives you the information, and you implement it*.” [Participant # 4].

##### 3.2.1.2 Theme 2: negative expectations

Most patients who participated in the study perceived the importance of the dietitian’s role and the benefits of nutrition counseling sessions. Nonetheless, some participants did not expect any benefits from nutrition counseling and reported they would rely on physical activity instead. A participant who had not visited the dietitian in the past 6 months said, *“I visited [the dietitian], but it’s nonsense. She [the dietitian] tells you to eat a cucumber, and then you gather the meat and rice from one end of the table to another. Our reality is that we live in a hot country. That’s the problem. I have worked in many countries. We walk a lot, and I don’t even take my medications when I am out of the country. Maybe when I don’t walk, my blood sugar rises a little.”* [Participant # 17].

The interviews also indicated some of the participants’ lack of awareness about the role and importance of a dietitian, despite a recent visit: “*I did [visit her], but I do not know her exact role. I know I need to maintain my blood sugar, and I have memorized some things that will help me.”* [Participant # 16].

One respondent expressed the belief that visiting the dietitian was associated with becoming ill, stating, *“The day I go to them, they give me the eye. I went to the clinic the last time, and after the visit, I had a fever for a week and a cough. Once I visited them, I became sick and fell ill. Even if you tell me to go to them, I will never go back. I decided that if I have an appointment with a dietitian, I will not attend.*” [Participant # 32].

The results of this study revealed the intricate relationship between mental well-being and the perceived significance of dietitian consultations. One of the participants who was referred to the dietitian but did not attend the session, stated: *“It [nutrition counseling] is beneficial if the person is feeling stable and mentally comfortable* [. ….]. *Today, I had an appointment. They asked me if I wanted to see the dietitian, but I did not want to because it added pressure.”* [Participant # 10].

External factors, such as work-related stress, interfered with the patient’s desire to prioritize dietitian consultations. This was reflected in the following statement: *“It is beneficial if the person is feeling stable and mentally comfortable, but I am currently under pressure in terms of work and obligations… A nutritionist can organize your life. Sure, she tells you, no, for sure, she explains to me that you are supposed to eat such and such quantities, the problem is that I do not hear the words*.” [Participant # 10].

Several participants acknowledged prior knowledge of healthy dietary practices but struggled to adhere to dietitian recommendations consistently, highlighting the challenge of bridging the gap between awareness about healthy practices and behavioral changes. One respondent said: *“I know about eating whole grains, reducing sugar, avoiding harmful food, sports, and walking. I know this information already….”* [Participant # 5].

#### 3.2.2 SCT social support

##### 3.2.2.1 Theme 3: social support as a motivator and enabler

The results of this study emphasize that social support is a valuable enabler and motivator for patients with diabetes to visit dietitians and implement nutritional recommendations effectively. Participants cited receiving support from health professionals, family members, and friends.

###### 3.2.2.1.1 Sub-theme 3.1 supportive healthcare professionals

The healthcare professional-patient relationship and support were evident in the participants’ descriptions of interactions with their doctors. Participants reported that doctors provide treatment plans and medication prescriptions and motivate them to have regular follow-ups with dietitians. This perspective was reflected in the following statement: *“The attending doctor is the one who motivates me. She is the one who checks what I am doing. […], for example, when I became pregnant, I was terrified of the issue, but the doctor said I want you to take the medicine and be committed to following an organized nutrition plan. I would like you to go to a special doctor for diabetes patients.”* [Participant # 9].

Participants also cited the supportive role of dietitians: *“The nutritionist encourages me to visit her and attend appointments.”* [Participant # 2].

###### 3.2.2.1.2 Sub-theme 3.2 supportive family and friends

Analysis of the interviews revealed that having supportive family and peers was crucial to diabetes self-management. Participants commented that family members and friends actively advise and encourage them to follow healthy eating habits and engage in physical activities: *“They influence you 100%. I have daughters, they monitor me and support me. At work, because of my relationship with them, they are like my sisters; if I did not bring my ‘dawah” [medicine], they bring it. They want me to get better and control my sugar, especially since I have type 2 diabetes, because I suddenly became obsessed with eating, and my weight has increased since my childhood. I have suddenly gained more than 20–30 kilos in the past few years. They are supporting me, but the problem is that I am moody. They are all trying to help me with this thing.*” [Participant # 10].

*“The family, if you follow a diet, they will provide you with everything you want, they will motivate you and give you support to do this and that.”* [Participant # 27].

Another respondent said: “It is important to be supported by family and friends; one hand doesn’t clap. I like it when my son alerts me and says, “A lot of dates are harmful to you, mother.” He is protective and cares about me.” [Participant # 5].

Two areas of support received from family and friends were related to emotional influence, accountability, and reminders.

*Emotional influence 3.2.1*: Participants highlighted the emotional aspect of social support. They mentioned that emotional bonds with family members motivate patients to make health-conscious choices. The following quote is from a participant who was referred to a dietitian but did not attend the visit. It highlights the emotional connection that influences individuals to adhere to advice from their social circles.

*“They are supporting me, but the problem is that I am moody. They are all trying to help me with this thing.*” [Participant # 10].

*Accountability and reminders 3.2.2*: Accountability and reminders emerged as key issues that enhanced participants’ commitment to following the dietitian’s recommendations. Participants cited that their family and friends are accountability partners, monitoring their dietary choices and encouraging healthy behaviors, as illustrated by the following statement: “*They say, Mother, do not eat anything you want with sugar. It is not good. Eat this and eat this. My daughters know, and they told me.”* [Participant # 23].

##### 3.2.2.2 Theme 4: participant suggestions

###### 3.2.2.2.1 Sub-theme 4.1 visiting a dietitian

Interview participants recommended that patients visit a dietitian and cited various benefits of dietitian nutrition counseling:

*“Tell them [patients] that they should go on the right path if they want good health, vision, sight, and hearing”.* [Participant # 23].

*“I advise them that this is something for their health. Of course, if it were not for a nutrition specialist after the doctor, I would neglect that. I would rely on the medicine only, and I do not want to rely entirely on it. I also have to rely on myself, organizing my health, my eating, and my physical activity. The dietitian is very important*.” [Participant # 21].

*“Yes, it helps them, and a visit is a must. If you have diabetes, you must visit the dietitian, even if it is once, twice, or three times. It is not necessary for each visit. Anyone who tells you about a specific topic and has a more profound knowledge will benefit you, whether it is in the near future or not. If you are knowledgeable about a particular topic, you would be able to help me. The dietitian is a specialist in her area. She will benefit you. Yes, I confirm that her words to the patient are correct and the nutritional program is accurate and benefits us.*” [Participant # 25].

###### 3.2.2.2.2 Sub-theme 4.2 changing lifestyle

Participants in the study made several suggestions for other patients to change their lifestyles for diabetes management, such as eating earlier in the evening, avoiding sugary foods, and engaging in physical activity. These perspectives are reflected in the following participant statements:

Suggestion on avoiding too much food and eating late in the evening: “I cannot tell them [patients] anything. One is in control of the decisions. For example, do you really have to eat until you are full and then sleep? You will have bacteria in your stomach, which can cause problems even in young ones, for example, having a late dinner after Maghrib prayer. If “Isha” prayer comes, my advice is don’t have dinner, and that’s it.” [Participant # 17].

Suggestion on eating less sugar: “I will tell them [patients] to stop eating sweets in the form of sugar, and in the form of milk containing sugar, they should cut off sugar. The food now is mostly sugar, biscuits, this, and everything that is prepared with sugar. A person should hold his hand a bit, and he shouldn’t eat anything if he wants his health.” [Participant # 23].

Suggestion on organizing personal life: “I think that when someone has diabetes, the first thing that they should do is organize their life, food, sleep, and physical activity. Everything. After that will come the medication.” [Participant # 36].

## 4 Discussion

This study explored patients’ attitudes toward dietitian-led nutrition counseling, focusing on the outcome expectancies and social support constructs of Social Cognitive Theory (SCT) within the cultural context of the UAE. SCT provides a useful framework for understanding the mediators of behavior change (43). Through qualitative interviews, patients’ perceptions of dietitians and the perceived outcomes of nutrition counseling for diabetes management were explored. The findings revealed mixed perceptions: some participants anticipated positive outcomes from dietitian visits, while others expressed skepticism.

Previous studies have shown that social support from family members, healthcare providers, and broader socio-cultural factors influences patients’ engagement in diabetes self-management behaviors (21, 44, 45). Evidence from the Arab Middle East further supports the relevance of SCT, demonstrating that social support, cognitive factors, and cultural norms shape adherence to diabetes self-management and lifestyle interventions (46–51). These findings align with the present study, highlighting the importance of outcome expectancies and social support within the sociocultural context of the Arab Gulf region.

In the present study, participants identified positive outcome expectancies as a major motivator for dietary adherence. Perceived benefits, such as improved glycemic control, better health outcomes, and weight management, reinforced adherence to dietitian recommendations. This finding is consistent with a hospital-based cross-sectional study conducted in Ethiopia (19), which found that healthcare professionals’ provision of dietary information was a predictor of good dietary practices.

Patients in this study perceived that dietitians, owing to their extensive expertise, can provide essential dietary information and correct misconceptions. This supports the findings of a cross-sectional study in Lebanon that assessed the frequency and determinants of use of dietary counseling services among patients with diabetes and highlighted patients’ belief in the usefulness of dietitian-led nutrition counseling (17). Another study that incorporated thematic analysis in its qualitative research revealed that patients appreciated the detailed nutrition advice provided by dietitians (18).

Despite these enablers, participants in the present study also reported barriers to adherence to dietary recommendations, including skepticism about dietary advice. Participants expressed hesitation about implementing recommended dietary changes, citing misinformation about the role of dietitians in diabetes management. Some participants reported an initial lack of understanding of dietitians’ functions and expertise, which hindered their engagement in nutrition counseling. This finding is consistent with Masupet et al. (16), who found that knowledge gaps about healthcare roles can limit active participation among patients with diabetes in South Africa. Similar findings have also been reported in studies conducted in Australia (20, 52). Misconceptions about diets and the role of dietitians were among the major barriers affecting patients’ decisions to seek nutrition counseling (20). In a qualitative study, dietitians themselves cited patients’ limited knowledge about the roles of diet and dietitians as the potential reason for patients with diabetes not seeking dietetic consultations (52). Therefore, educational programs aimed at raising awareness of dietitian-led interventions can bridge this gap and emphasize the benefits of MNT in achieving better glycemic control (13, 14).

Among other negative expectations presented in this study, some patients perceived that visiting a dietitian was not useful, as they felt they already had sufficient knowledge. This could make it difficult to bring about behavioral change. A previous study also identified patients’ perceptions that they already possessed enough knowledge as a possible explanation for their reluctance to seek nutrition advice (52). This study also indicated that patients may avoid dietetic consultations because they fear judgment and blame from healthcare providers for their poor choices, underscoring the importance of empathy and positive support from healthcare professionals (52).

Some participants expressed skepticism toward dietary advice when it appeared to conflict with culturally familiar eating practices, such as traditional meals centered around meat and rice. These responses highlight how dietary recommendations perceived as incompatible with established cultural food patterns may reduce patients’ willingness to engage in nutrition counseling. A study by Atomei et al. (53) emphasizes the importance of cultural competence in dietetic practice. Tailoring nutrition interventions and counseling to the cultural diversity of regions improves health parameters and patient satisfaction (53). In addition, several participants described dietitian visits as an “added pressure” during periods of emotional stress or competing life responsibilities. These perspectives illustrate how psychological burden and daily life demands can influence patients’ readiness to seek or implement dietary guidance. When interpreted through the lens of SCT, such experiences reflect negative outcome expectancies and perceived barriers that may weaken motivation to adopt recommended dietary behaviors. Understanding these perceptions within the cultural and social realities of patients’ lives is essential for designing nutrition counseling strategies that are both culturally sensitive and responsive to patients’ lived experiences.

Social support emerged as a key facilitator, with participants in the present study emphasizing the role of family and friends in encouraging healthier food choices and physical activity. Similar findings have been reported in a study conducted in Saudi Arabia that examined the influence of social support on diabetes self-care behaviors using the Multidimensional Scale of Perceived Social Support (MSPSS), in which family members were identified as the primary source of support among patients with diabetes (54). Vahedparast et al. and Wichit et al. also reported that strong social networks mitigate isolation and distress, providing both emotional reinforcement and practical assistance (21, 22). Social support also enhances self-efficacy, a critical component of SCT, which promotes sustained behavior change (15, 28). The positive influence of social networks on chronic disease management highlights their role in reinforcing adherence to nutrition counseling and encouraging healthier lifestyle choices. On the other hand, a lack of social support can negatively impact diabetes self-care (55). Therefore, diabetes management in nutrition counseling should involve patients’ families so that they are well-equipped to provide behavior change support to their family member (44).

Participants in this study highlighted the motivating role of physicians in encouraging their engagement with dietitians. This supports the findings of previous studies advocating multidisciplinary care models (20, 36). A medical practitioner’s referral to a dietitian motivated patients to seek nutrition advice. This finding is in line with a study conducted in Lebanon (17). A study by Siopis et al. in Australia found that in the absence of such referrals, patients’ access to dietary advice is hampered (52). Moreover, inter-professional collaboration, in which dietitians collaborate with physicians and nurses, ensures consistent messaging and enhances patient-centered diabetes management.

Viewing participants’ perspectives through the SCT constructs of outcome expectancies and social support, within a culturally responsive lens, highlights how patients interpret, and sometimes resist, dietary recommendations in the Arab Gulf context. These qualitative insights provide a deeper understanding of the contextual and experiential factors shaping behavior, which are often not captured by quantitative measures of dietary adherence.

Participants in the study made several suggestions to other patients with diabetes to follow dietitians’ nutrition advice, including food types, meal timing, and increased physical activity. These suggestions can be considered for incorporation into future culturally competent supervised peer education and support groups for people with diabetes in the UAE.

The analysis of participant responses reveals diverse perspectives on the importance of dietitian consultations, a demand for comprehensive guidance, and obstacles in translating awareness into actionable habits. These findings underscore the need for diabetes management clinics to go beyond offering dietary advice alone. Adopting a holistic approach that includes mental well-being, tailored guidance, and practical strategies for fostering behavioral change can improve adherence to recommendations and enhance diabetes management outcomes. Encouraging family involvement and raising awareness about the benefits of dietitian-led interventions should be considered as additional strategies to enhance adherence.

Research from the Middle East shows that diabetes management extends beyond traditional nutrition counseling to include multicomponent lifestyle interventions combining diet, physical activity, behavioral support, and self-management education, which have improved glycemic control and cardiometabolic outcomes (56, 57). Culturally adapted approaches, such as the Transcultural Diabetes Nutrition Algorithm and community-based education programs, further enhance adherence to healthy behaviors and clinical outcomes in the region (58, 59).

### 4.1 Strengths

The strengths of this study lie in its qualitative approach, which provided in-depth insights into patients’ perspectives, beliefs, and experiences in a culturally unique setting in the Arab Gulf region. It is also the first study to explore patients’ perspectives on SCT’s social support and outcome expectancies in the Middle East and North Africa (MENA) region. Furthermore, we used concurrent data collection and analysis to identify the preliminary codes and categories from the data after the first 22 interviews. The recurrence of these codes and categories from the whole sample of 44 participants confirmed data saturation. It strengthened the credibility of the findings despite the relatively short duration of the individual interviews.

### 4.2 Limitations

The main limitation of this study is that it was conducted in a single healthcare setting and included only UAE nationals, which may limit the transferability of the findings. Consistent with qualitative research, the results of this study are not intended to be generalizable. However, the inclusion of 44 interviews enhances the diversity of perspectives, and credibility was supported through purposive sampling, independent coding, and data saturation. Future research should include more diverse settings and incorporate quantitative measures to examine the relationship between patients’ perceptions and clinical outcomes in diabetes.

## 5 Conclusion

The study results highlight the importance of social support and positive outcome expectations as key enablers of dietary adherence. Social support from physicians, family members, and peers influenced patients’ motivation, accountability, and adherence. However, participants’ experiences varied depending on individual circumstances, social contexts, and barriers such as skepticism and limited awareness of dietitians’ roles. These findings illustrate how the SCT constructs of outcome expectancies and social support are embedded within the lived experiences of patients managing diabetes in the cultural context of the Arab Gulf region.

Tailored interventions and strengthened support systems can improve adherence to dietitian recommendations. Participants’ mixed perceptions of dietitian-led nutrition counseling underscore the need for improved communication and culturally tailored interventions. Future research should examine the influence of socio-cultural factors on diabetes self-management outcomes in the UAE.

### 5.1 Implications for practice

The results of this study have implications for enhancing patients’ dietetic services and promoting their engagement with diabetes self-management education. Diabetes management clinics can improve outcomes by fostering trust between patients and dietitians, increasing motivation, enhancing social support, and addressing informational barriers to encourage better patient engagement and adherence to diabetes nutrition care.

## Data Availability

The original contributions presented in the study are included in the article/Supplementary material, further inquiries can be directed to the corresponding author.
