# Supplementary material for: The role of outcome expectancies and social support in adherence to nutrition counseling: perspectives of Emirati adults with diabetes
Source: Front Public Health. 2026 Apr 16;14:1805632. doi: 10.3389/fpubh.2026.1805632 (PMC13128802; doi:10.3389/fpubh.2026.1805632)
Supplement: Supplementary file 1 [file Supplementary_file_1.pdf]

Supplementary Material

Supplementary material 1: Interview discussion guide

Project Title: **Perspectives of Patients with Diabetes on Accessing Nutrition Counseling**

Interview Discussion Guide: Outcome Expectancies and Social Support

Introduction:

Thank you for your willingness to participate in this interview.

Patients are often referred by their doctors to the center dietitian for nutrition counseling. The purpose of this interview is to get your opinion on topics related to nutrition counseling for patients with diabetes. I would like to get your opinion about the benefits of visiting the dietitian for nutrition counseling, what motivates you to see the dietitian, and your suggestions for motivating patients to attend their scheduled appointments with the center dietitian.

I am audio-taping this discussion so that the information we collect is as complete as it can be. Your name will not be attached to the taped discussion or any reports from the study.

- Please state your first name and how long you had diabetes.
- Let's start our discussion with what you see as the benefits or what motivates you to attend the dietitian appointment for nutrition counseling

Question 1: Do you believe that visiting a dietitian is important to improve your health, your diabetes, weight, etc.? Please explain

- If you think it is important to see the dietitian for nutrition counseling, can you tell me why it is important and if you think it is not important, please tell me what your reasons are

Question 2: What benefits do you think you may get by visiting the dietitian for nutrition counseling?

Managing diabetes requires time, effort and support from others, such as family, friends, and health professionals

Question 3: How do your family and friends influence your eating habits or physical activity to manage your diabetes?

Question 4: Who motivates you to have regular follow ups with the dietitian: dietitians, doctors, family members, friends? What do they normally say?

Question 5: What kind of help do you need from your dietitian to overcome any difficulties that you have in following the recommended changes in your food and physical activity?

- Who else do you think can help?

Question 6: What do you suggest to have patients become more motivated to apply the recommendations of the dietitians in their daily lives, such as changing what they eat or to becoming more physically active?

Closing: Do you have anything else you would like to add to our discussion about visits to the dietitian or nutrition counseling?

Thank you for the opportunity to share your opinion with us.
